# Physiological Strabismus

**Published:** 1893-05

**Authors:** Julius Pohlman

**Affiliations:** Buffalo, N. Y.


### PHYSIOLOGICAL STRABISMUS.

By JULIUS POHLMAN, M. D., Buffalo, N. Y.

Dr. Pohlman asked to be forgiven for the illogical title, as he would rather have called his paper: Shall We Teach Our Children How To Squint? From a series of examinations he had found reason to conclude that the power of convergence of the eyes did not agree with the power of accommodation as regularly as our textbooks teach. He had, so far, found limited convergence in cases of syphilis, alcoholism, and after scarlet fever, with otherwise evenly balanced eyes, and asked the members to aid him with additional observations and explanations on this subject; and if it was finally found that a variety of causes did produce limited convergence in the adult, then it may be worth while to ask the question: Shall we teach children how to squint?

Discussion of Dr. Pohlman's paper:

Dr. A. A. Hubbell: The doctor asks why all persons do not converge to an extraordinary degree. To this I will reply that it is not necessary. To have children practise convergence is, to my mind, bad policy, and leads to permanent squint. I do not yield to Dr. Pohlman on this point. If the child did possess an unusual power to converge the eyes, and practised it, it would surely lead to a permanent result, and, therefore, this practice should be discouraged.

Dr. F. W. Abbott: As to the sanitary effects of straining the muscles of the eyeball, it is very strange to me, and I agree with Dr. Hubbell that it is a bad practice, and is, therefore, unnecessary. The relation between the faculty of accommodation and convergence is one that we pay considerable attention. For easy vision we should be able to accommodate to a point a little nearer than we are obliged to, and also to a point a little further than necessary. I do not see the need of straining the power of convergence or of accommodation.

Dr. J. W. Putnam: Upon the proper balance between the external and internal recti muscles depends in a great measure single vision. The doctor, by encouraging his patients to converge their eyes, exercises the internal recti muscles only. If he also encourages exercise or gymnastics of the external recti muscles, I can see no danger in this proceeding.

Dr. L. Howe: Donders and Landolt have devoted much time to this subject. The power of convergence varies with age. We must all admit that as far as the muscles of the eye are concerned, we are very much at sea. Dr. Pohlman should classify his cases.

Discussion of Dr. Dagenais' paper:

Dr. J. H. Pryor: Did you notice the number of respirations per minute? A. Fifty to sixty.

Dr. M. HARTWIG: Was the case delivered with forceps? A. No. If there was no opening of the foramen ovale, then I would consider the case one of syphilis.

Dr. DAGENAIS: There was nothing to indicate syphilis, nor any history of the disease.

The discussion on Dr. Dunham's paper was postponed to a later meeting.

Pathological specimens were presented by Drs. Pryor and Krauss, Mynter, and McAdam and Krauss.

Cases were reported by Drs. Crego, Mynter, and Hartwig.

